# Integrins and cadherins as therapeutic targets in fibrosis

**DOI:** 10.3389/fphar.2014.00131

**Published:** 2014-06-03

**Authors:** Sandeep K. Agarwal

**Affiliations:** Section of Allergy, Immunology, and Rheumatology, Department of Medicine, Biology of Inflammation Center, Baylor College of Medicine, Houston, TX, USA

**Keywords:** integrins, cadherins, fibrosis, macrophage, fibroblasts, epithelial cells

## Abstract

Fibrosis is the excessive deposition of extracellular matrix proteins into tissues leading to scar formation, disruption of normal tissue architecture and organ failure. Despite the large clinical impact of fibrosis, treatment options are limited. Adhesion molecules, in particular αvβ6 and α3β1 integrins and cadherin-11, have been demonstrated to be important mediators of tissue fibrosis. These data are reviewed here and provide the foundation for these molecules to be potential therapeutic targets for patients with fibrotic diseases.

## Introduction

Fibrosis is the excessive deposition of extracellular matrix proteins into tissues leading to scar formation, disruption of normal tissue architecture and organ failure (Wynn, 2008; Wei et al., 2011a). Fibrosis is the final common pathway of a tissue's response to injury, including chemical exposures, infections, and autoimmunity. Multiple tissues in the body can be affected by fibrosis, with the majority of research focusing on lung, kidney, liver and skin fibrosis. The overall clinical impact of fibrosis is large, with some reports suggesting that up to 45% of deaths in the industrial world can be attributed to fibrosis (Wynn, 2008). Despite its importance, current treatment options are extremely limited and largely supportive rather than curative. Pirfenidone has shown promise in the treatment of idiopathic pulmonary fibrosis (Noble et al., 2011) and other targets are at various stages of development (Chakraborty et al., 2014). However, the clinical need for therapeutic interventions in fibrotic diseases is still clearly present. Therefore, understanding the cellular and molecular pathways that lead to the development of tissue fibrosis is critical to identify potential therapeutic targets

Current paradigms indicate that epithelial cells, macrophages, T-cells, and fibroblasts all contribute to the development of tissue fibrosis (Wynn, 2008; Wei et al., 2011a). Injured epithelial cells become reprogrammed, initiating and propagating mesenchymal pathways, most notable of which are TGFβ related pathways (Camelo et al., 2014). Macrophages also contribute to the early inflammatory process and subsequently the fibrotic process through the production of profibrotic cytokines and growth factors, including TGFβ, that recruit and activate fibroblasts (Denholm and Rollins, 1993; Atabai et al., 2009; Pesce et al., 2009; Mathai et al., 2010). Ultimately these processes lead to the accumulation of activated fibroblasts and myofibroblasts at the sites of fibrosis. Myofibroblasts are key producers of the extracellular matrix that is laid down during development tissue fibrosis (Hinz et al., 2007; Wynn, 2008). The collagen and extracellular matrix deposition replaces the normal tissue architecture which in turn leads to organ dysfunction. The source of fibroblasts and myofibroblasts is very controversial and potentially includes resident fibroblasts, bone marrow derived mesenchymal precursors (fibrocytes), and epithelial cells [via the process of epithelial-to-mesenchymal transition (EMT)] (Kim et al., 2006, 2009a; Tanjore et al., 2009; Degryse et al., 2010; Chapman, 2011; Rock et al., 2011). The relative contributions of three potential sources may differ depending on the underlying stimulus, the tissue undergoing fibrosis and the mouse model of fibrosis. Regardless of the controversy over cellular origins, the molecular pathways that govern these processes are similar. TGFβ is considered to be the central mediator of fibrosis, but the cytokines (IL4, IL-13, IL-6), chemokines (CCL2), PDGF, Wnt signaling pathway, and beta-catenin, also contribute to the fibrotic response (Varga and Pasche, 2009; Varga and Whitfield, 2009; Lam et al., 2011; Wei et al., 2011b, 2012). In the current review, we will discuss how the cellular adhesion molecules, namely the integrins and cadherins, may contribute to the development of tissue fibrosis.

## Cellular adhesion molecules

The ability of cells to adhere to each other and to interact with the extracellular matrix through cellular adhesion molecules is important in regulating a variety of biological processes including tissue remodeling and inflammation. Cellular adhesion molecules have been classified into four families (selectins, immunoglobulin superfamily, integrins, and cadherins) based on their molecular structure. The current review will only focus on integrins and cadherins as they relate to the development of tissue fibrosis. A more detailed discussion of each family can be obtained in several excellent reviews (Springer, 1994; Petruzzelli et al., 1999; Hynes, 2002; Patel et al., 2002; Wheelock and Johnson, 2003a; ffrench-Constant and Colognato, 2004; Humphries et al., 2006).

Integrins are a large family of transmembrane adhesive proteins that influence a wide array of biologic processes including tissue organization and inflammation (reviewed in Hynes, 2002; ffrench-Constant and Colognato, 2004). Integrins are heterodimeric glycoproteins consisting of an alpha- and a beta-chain. Each subunit contains a long extracellular domain, a transmembrane region and cytoplasmic domain capable of connecting to the actin cytoskeleton and triggering signal transduction events into the cell. There are currently 18 alpha subunits and 8 beta subunits, which can combine to form 24 different integrin heterodimers. Integrins can be further classified into: arginine-glycine-aspartate (RGD) binding integrins, the α-4 integrins, leukocyte adhesion integrins, laminin-binding integrins, and I-domain collagen binding integrins. On the extracellular surface, integrins can interact with other adhesion molecules, such as the immunoglobulin superfamily members and cadherins, growth factor receptors, and the extracellular matrix. In addition to linking the extracellular environment of the cell to the actin cytoskeleton, the cytoplasmic tail of integrins also serves as an anchor for a large number of signaling molecules. Therefore, integrins are capable of regulating cell behavior through a number of different pathways and mechanisms. As a result, integrins are involved in a large spectrum of human health and diseases, including thrombotic, infectious, malignant, and inflammatory diseases (Goodman and Picard, 2012). We will discuss the importance some of the integrins in the fibrosis.

Cadherins are a family of adhesion molecules that mediate homophilic, calcium-dependent cellular adhesion by binding a cadherin of the same type on an adjacent cell (homophilic adhesion) (Takeichi, 1990; Wheelock and Johnson, 2003a; Goodwin and Yap, 2004). Classical cadherins possess five extracellular domains, a single pass transmembrane domain and a highly conserved cytoplasmic tail (Boggon et al., 2002). The cytoplasmic tail interacts with beta-catenin, which in turn binds alpha-catenin, forming a linkage between the cadherin-catenin complex and the actin cytoskeleton (Horikawa et al., 1999). The spatiotemporal expression pattern of cadherins during embryogenesis is critical in cell migration, cell differentiation and tissue morphogenesis. In the postnatal environment, cadherins play a role in the maintenance of tissue architecture (Hermiston and Gordon, 1995). Cadherins have functions that extend beyond cell-to-cell adhesion. Cadherins are linked to multiple intracellular signaling pathways, including WNT, PI-3 kinase/Akt and FGF pathways (Nakagawa and Takeichi, 1998; Suyama et al., 2002; Tran et al., 2002). Furthermore, cadherins have been implicated in malignant transformation and tumor invasiveness (Shibata et al., 1996; Hazan et al., 1997, 2000; Pishvaian et al., 1999; Tomita et al., 2000; Wheelock et al., 2001). During the process of malignant transformation and EMT, epithelial cells become more invasive, which is associated with the down-regulation of E-cadherin and up-regulation of mesenchymal cadherins such as N-cadherin and cadherin-11 (Hazan et al., 1997, 2000). Finally, cadherins have also been implicated in regulating inflammation and cartilage damage in mouse models of inflammatory arthritis (Lee et al., 2007; Park et al., 2011). Together, these studies demonstrate an important role for cadherins in health and diseases. In the current review, we will discuss recent studies implicating cadherins in the pathogenesis of fibrosis.

## Alpha-V integrin regulation of TGFβ during fibrosis

**Figure 1:**
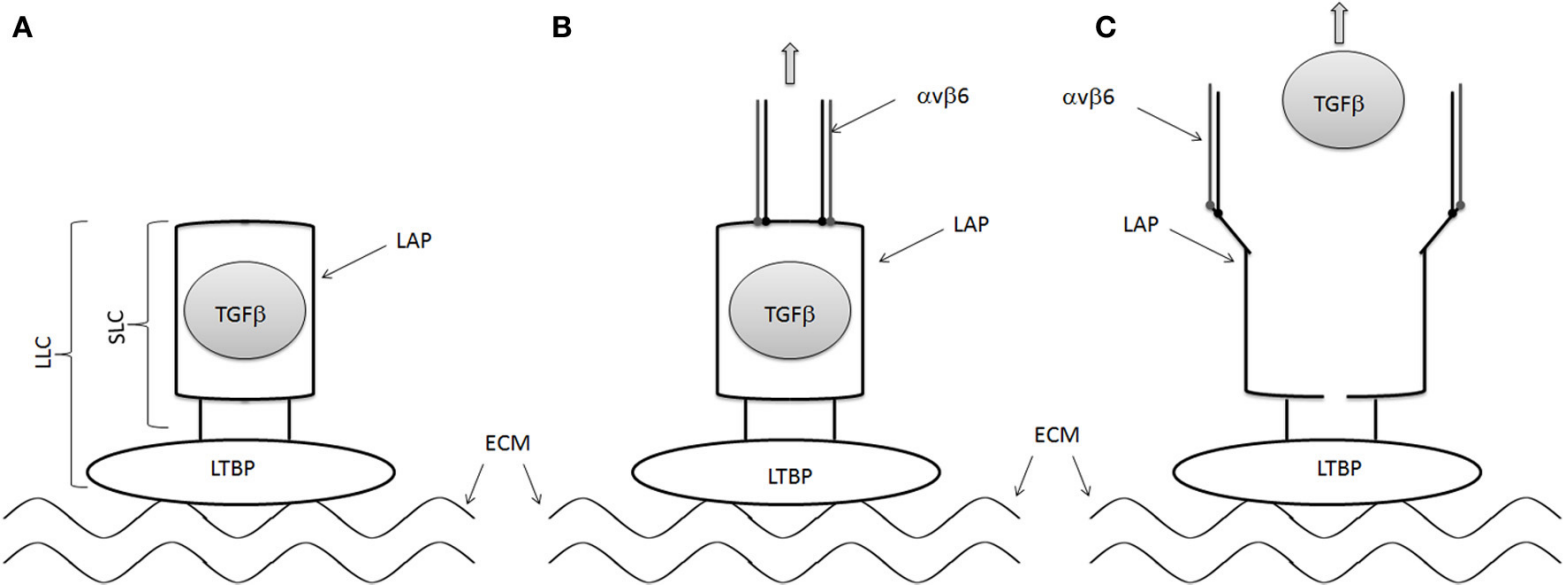
**Mechanisms of TGFβ activation by αvβ6 integrin. (A)** LAP forms a straightjacket around TGFβ in the small latent complex (SLC) and binds to LTBP to for the large latent complex (LLC). The LTBP is in turn tethered to the extracellular matrix. **(B)** The cell associated αvβ6 integrin binds to the RGD domains of LAP, pulling the LAP ope against the resistance provided by the extracellular matrix/LTBP interaction. **(C)** TGFβ is then released from the LAP, allowing it to interact with its receptor.

TGFβ is one of the key growth factors involved in the fibrotic process. There are three isoforms of TGFβ (TGFβ1, −2, −3). The gene for TGFβ encodes a precursor protein, consisting of a C-terminal TGFβ molecule and an N-terminal region that encodes a protein called the latency-associated peptide (LAP). The precursor protein is cleaved followed by a noncovalent association of these peptides to form the small latent complex (SLC, Figure 1A). The SLC subsequently associates with latent TGFβ–binding proteins (LTBP) in the extracellular matrix to form the large latent complex (LLC, Figure 1A). TGFβ in the context of the LLC is secreted from the cell and remains hidden in its inactive state in the extracellular matrix. Activation of TGFβ in the extracellular matrix is required for TGFβ to activate its receptors and is thought to be an important regulatory step in the development of fibrosis. Changes in pH, changes in temperature, reactive oxygen species and protease activation by plasmin, thrombospondin 1 may activate TGFβ.

The effects of TGFβ are pleiotropic and can vary significantly depending on the context within which it is expressed. During the fibrotic process, TGFβ1 promotes collagen production by fibroblasts, can induce EMT and myofibroblast differentiation, and cellular migration. However, TGFβ1 deficient mice develop diffuse, multifocal mononuclear inflammation resulting in mortality due to cardiopulmonary complications (Shull et al., 1992). Therefore, despite its critical importance in fibrosis, systemic and direct inhibition of TGFβ in fibrosis may not be the ideal approach to the treatment of fibrosis. Rather indirect approaches, as discussed below, may allow more targeted inhibition of TGFβ in fibrosis.

RGD-binding integrins, including the αv integrins, have been reported to activate latent TGFβ (Munger et al., 1998, 1999). Human LAP-β1 and LAP-β2 contains an RGD motif, suggesting that they may interact with RGD-binding integrins. Indeed several αv integrins, including αvb1, αvb3, αvb5, and αvβ6 integrins, have been shown to bind to LAP-β1 and LAP-β2 (Munger et al., 1998, 1999). Furthermore, αvβ6 integrin has been shown to activate latent TGFβ The activation of TGFβ by αvβ6 is dependent on an intact cytoskeleton, implying that mechanical force generated by the integrin can alter the structure of the LAP in the SLP of TGFβ. A recent crystallographic analysis of the SLC of TGFβ confirmed this hypothesis (Figure 1) (Shi et al., 2011) These data suggest that LAP forms a straightjacket around TGFβ while binding to the LTBP via a disulfide bond. At the same time, the LTBP interacts with the extracellular matrix. The cell associated αvβ6 integrin binds to the RGD domains of LAP, pulling the LAP open against the resistance provided by the extracellular matrix/LTBP interaction, activating TGFβ by releasing it and allowing it to interact with its receptor.

The expression of αvβ6 integrin has been described on type II alveolar epithelial cells of patients with idiopathic pulmonary fibrosis and scleroderma lung fibrosis (Horan et al., 2008). Murine studies have demonstrated αvβ6 integrin expression is markedly upregulated by injury and inflammation in the lung as well (Munger et al., 1999). The central importance of alveolar epithelial cells in fibrosis has recently been reviewed (Camelo et al., 2014). Consistent with a potential role for αvβ6 integrin on epithelial cells in the development of fibrosis is the observation that β6 null mice are protected from lung fibrosis in the intratracheal bleomycin-induced lung fibrosis model, even though the β6 null mice suffer from an exaggerated pulmonary inflammatory response (Munger et al., 1999). These data were further confirmed using an anti- αvβ6 integrin antibody, which attenuated collagen expression and fibrosis in the intratracheal bleomycin-induced lung fibrosis model (Horan et al., 2008). In addition an important role for αvβ6 in the development of lung fibrosis has been demonstrated using β6 null mice and an anti- αvβ6 integrin antibody in the radiation-induced lung fibrosis mouse model and the TGFα induced lung fibrosis model (Puthawala et al., 2008; Madala et al., 2014). Finally, the role of αvβ6 integrin in renal fibrosis in the unilateral ureteral obstruction model and acute biliary fibrosis has also been shown, once again demonstrating commonalities of fibrosis in the different tissues (Ma et al., 2003; Wang et al., 2007).

The studies described above demonstrate an important role for the αvβ6 intergin in the development of tissue fibrosis. However, αvβ6 integrin may not be involved in all forms of fibrosis as noted by the lack of an effect in the carbon tetrachloride (CCL4)-induced liver fibrosis model. This may be due to the expression pattern of αvβ6 integrin or the ability of multiple beta integrin partners with the αv chain. It has been shown that myofibroblasts express αv integrins not associated with the β6 chain, several of which are capable of activating latent TGFβ. Accordingly, a recent study reported that αv integrin depletion in liver hepatic stellate cells and myofibroblasts using the PDGF receptor Cre promoter and loxP flanked αv integrin, protected mice from CCL4-induced liver fibrosis (Henderson et al., 2013). These data were confirmed using a small molecule inhibitor of αv integrin. In addition, several studies have demonstrated expression of αvβ3 and αvβ5 integrins on dermal fibroblasts and in skin biopsies of systemic sclerosis patients, suggesting that multiple αv integrins, other than only the αvβ6 intergin, may play a role in skin fibrosis (Asano et al., 2005a,b, 2006a,b). Together these studies identify αv integrins as important mediators of tissue fibrosis through the activation of latent TGFβ and suggest that αv integrins may be therapeutic targets in patients with tissue fibrosis.

## Integrin-cadherin cross-talk in fibrosis

Injury to the alveolar epithelial cells is a key event in the development of lung fibrosis (Camelo et al., 2014). As discussed above, epithelial damage activates TGFβ through αv integrins. TGFβ then drives fibrosis though activation of fibroblasts and myofibroblast differentiation. Whether epithelial cells are also directly a source of myofibroblasts in the fibrotic tissue, through the process of EMT, remains controversial. Inhibition of EMT has been suggested as a therapeutic strategy but it remains unclear if the success of EMT inhibition observed *in vitro* translates to the *in vivo* (Wang et al., 2010; Jang et al., 2013). However, epithelial cells in fibrotic lungs activate mesenchymal gene programs, including the Wnt signaling pathway and β-catenin (Chilosi et al., 2003; Konigshoff et al., 2008). Consistent with this, inhibition of Wnt and β-catenin signaling decreases the development of tissue fibrosis (Bayle et al., 2008; Bergmann et al., 2011; Beyer et al., 2012; Wei et al., 2012).

Beta-catenin regulates cell-to-cell adhesion and gene transcription through its interactions with cadherins and Wnt signaling. In the absence of Wnt signaling, β-catenin is serine phosphorylated by glycogen synthase kinase, which then designates it for proteosomal degradation. If the Wnt pathway is activated, β-catenin translocates to the nucleus and activates gene transcription. Beta-catenin also regulates cell-to-cell adhesion through binding to a highly conserved region on the cytoplasmic tail of cadherins. This association is reduced by tyrosine phosphorylation of β-catenin, which in turn makes β-catenin available to join the cytoplasmic pool and translocate to the nucleus to regulate gene transcription (Roura et al., 1999). Thus, a balance between the cytoplasmic and the cadherin-associated pools of β-catenin exists. Interestingly, TGFβ may alter this balance and has been shown to increase β-catenin mediated signaling in the nucleus (Masszi et al., 2004).

More recently, the α3β1 integrin has also been shown to regulate the β-catenin pool through interactions with E-cadherin (Kim et al., 2009a). Immune fluorescent studies demonstrated that α3β1 integrin co-localizes with E-cadherin. Biochemical studies in alveolar epithelial cells also have demonstrated that α3β1 integrin physically associates with E-cadherin and the TGFβ receptor (Kim et al., 2009a,b). This tri-molecular complex of α3β1 integrin, E-cadherin and the TGFβ receptor results in the tyrosine phosphorylation of β-catenin at position Y654, but not serine phosphorylation. pY654 β-catenin subsequently associates with pSmad2 (a TGFβ signaling molecule) and then translocates to the nucleus where it can regulate transcription. Interestingly, these pY654 beta-catenin-pSmad2 complexes have been identified in mouse fibrotic lungs as well as fibrotic lungs from idiopathic pulmonary fibrosis patients but not lungs from healthy or emphysematous patients. These data suggest a role for α3β1 integrin in the development of lung fibrosis through interaction with E-cadherin and downstream β-catenin signaling.

Support for this hypothesis has come from both *in vitro* and *in vivo* studies. *In vitro* studies have demonstrated that alveolar epithelial cells from α3 integrin chain deficient mice have decreased mesenchymal gene expression when plated on fibronectin, a known inducer of EMT (Kim et al., 2009a,b). Most important are the *in vivo* studies, which demonstrated that mice with selective loss of expression of the α3 integrin chain in epithelial cells have attenuated lung fibrosis in the intratracheal bleomycin lung fibrosis model (Kim et al., 2009a). Together these data support a role for the α3β1 integrin in fibrosis (likely through its interaction with cadherins and β-catenin), and suggest that α3β1 integrin targeting may be a therapeutic option in fibrosis.

## Cadherin regulation of fibrosis

Cadherins are important determinants of cell fate with important roles during both development and postnatally (Wheelock and Johnson, 2003a,b). E-cadherin is expressed on epithelial cells while other cadherins, including N-cadherin and cadherin-11, are expressed on mesenchymal cells including fibroblasts. Changes in the expression patterns of cadherins are an important determinant in the phenotype of malignant cells (Nieman et al., 1999; Pishvaian et al., 1999). Accordingly, the expression of cadherin-11 and N-cadherin and the loss of expression of E-cadherin confers a mesenchymal and invasive phenotype in breast cancer and other cancer cell lines (Hazan et al., 1997; Nieman et al., 1999; Pishvaian et al., 1999). EMT transition has been hypothesized to be one of the mechanisms that underlie the cadherin switch in these cells.

With regards to fibrosis, the role of cadherins has been indirectly considered and focused on E-cadherin, where its expression is lost on epithelial cells during the development of fibrosis (Tanjore et al., 2009). The increase in N-cadherin has also been tracked in alveolar epithelial cells hypothesized to be undergoing EMT (Tanjore et al., 2009). However, it is not clear from the literature if the changes in E-cadherin or N-cadherin are mechanistically involved in the development of fibrosis. More recently, cadherin-11, another mesenchymal cadherin, has been shown to be increased in fibrotic tissue and studied in the context of fibrosis (Schneider et al., 2012; Wu et al., 2014).

Cadherin-11 is a type-II mesenchymal cadherin initially identified in osteoblast cell lines but subsequently noted to be expressed in neural tissue, lung, and kidney (Okazaki et al., 1994; Hoffmann and Balling, 1995). Aberrant expression of cadherin-11 in malignant cells, including breast cancer and prostate cancer, is associated with a more invasive and metastatic phenotype (Shibata et al., 1996; Pishvaian et al., 1999; Tomita et al., 2000). Subsequent studies have also shown cadherin-11 expression on fibroblasts within the synovial lining of joints where it may regulate invasion and the production of interleukin-6 and matrix metalloproteinases (Lee et al., 2007; Chang et al., 2011; Noss et al., 2011). Interestingly, *in vivo* studies have demonstrated that cadherin-11 deficient mice have an attenuation in synovial inflammation in the KBxN serum transfer arthritis model, suggesting that cadherin-11 may be a therapeutic target for human inflammatory arthritis, such as rheumatoid arthritis (Lee et al., 2007).

More recently, a potential role of cadherin-11 in lung and dermal fibrosis has been postulated. This interest was stimulated by observations that cadherin-11 is expressed on multiple fibroblast populations, including dermal and lung fibroblasts, and two independent microarray studies of systemic sclerosis skin biopsies demonstrated increased cadherin-11 mRNA levels in fibrotic skin (Whitfield et al., 2003; Gardner et al., 2006). The increase in cadherin-11 expression in fibrotic tissue was subsequently confirmed using multiple techniques in systemic sclerosis skin biopsies and fibrotic lung tissue from patients with idiopathic pulmonary fibrosis (Schneider et al., 2012; Wu et al., 2014). Using murine models of skin and lung fibrosis, it is now evident that the increase in cadherin-11 in fibrotic tissue, is mechanistically involved in the development of fibrosis. Accordingly, cadherin-11 deficient mice have a significant decrease in dermal and lung fibrosis when challenged with subcutaneous or intratracheal bleomycin (Schneider et al., 2012; Wu et al., 2014). Importantly, anti-cadherin-11 monoclonal antibodies are also effective in treating existing fibrosis in these models as well (Schneider et al., 2012; Wu et al., 2014). These data suggest that the inhibition of cadherin-11 is a potential therapeutic strategy in patients with fibrosis.

**Figure 2:**
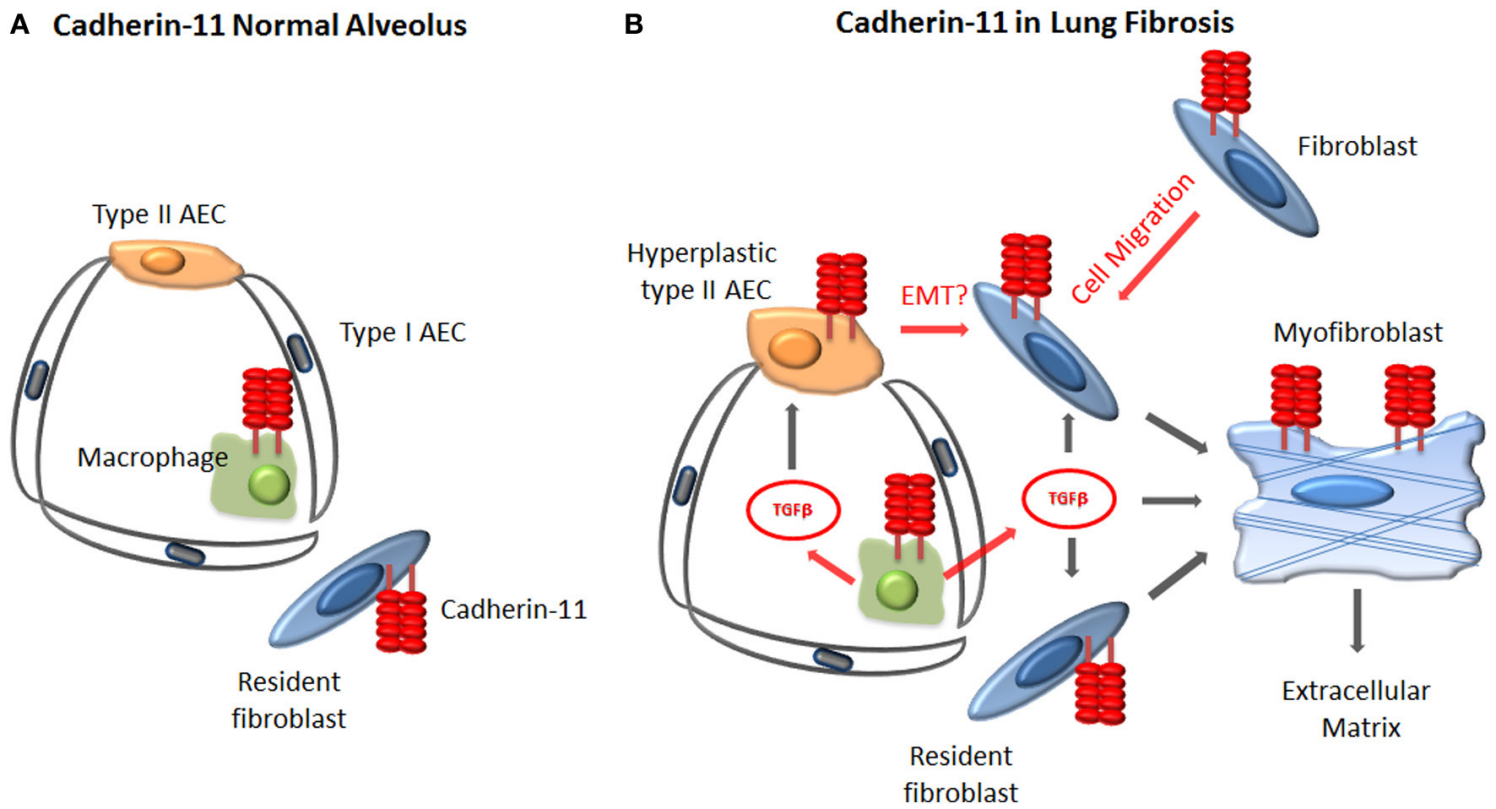
**Proposed cellular mechanisms by which cadherin-11 regulates fibrosis. (A)** In the healthy lung, cadherin-11 is expressed on the resident fibroblasts and at low levels on the alveolar macrophages. **(B)** During fibrosis cadherin-11 expression is noted on fibroblasts, myofibroblasts, alveolar macrophages, and injured type II alveolar epithelial cells. Cadherin-11 likely contributes to fibrosis through regulation of fibroblast migration, alveolar macrophage production of TGFβ, and mesenchymal gene expression and possibly EMT of type II AECs.

The mechanism by which cadherin-11 regulates fibrosis remains under investigation. Immunohistochemical studies have demonstrated that cadherin-11 is not only expressed on fibroblasts in fibrotic lung tissue, but also macrophages and hyperplastic type II alveolar epithelial cells (Schneider et al., 2012). In fibrotic skin, cadherin-11 was also confirmed on fibroblasts and macrophages (Wu et al., 2014). Given the expression of cadherin-11 on multiple cell populations involved in the development of fibrosis, it is likely that cadherin-11 modulates multiple steps of the fibrotic process, including the macrophage, epithelial cell and fibroblast (Figure 2). For example, cadherin-11 deficient macrophages produce less TGFβ, but similar amounts of TNFα, compared to wild type macrophages (Schneider et al., 2012; Wu et al., 2014). Furthermore, inhibition of cadherin-11 expression in lung epithelial cell lines blocked mesenchymal gene expression and EMT *in vitro* (Schneider et al., 2012). Finally cadherin-11 has been shown to regulate dermal fibroblast migration and the levels of β-catenin, both of which are important in the development of tissue fibrosis (Wu et al., 2014). Therefore, combined with the *in vivo* murine studies, cadherin-11 appears to be an important mediator of tissue fibrosis and inhibition of cadherin-11 function is a potential therapeutic approach for the treatment of tissue fibrosis.

## Conclusions

The understanding of the development of tissue fibrosis has greatly expanded in recent years. Many of these pathways are shared in the different tissues that develop fibrosis, including the lung, skin, liver, and kidney. The clinical implications of fibrosis are substantial, however to date disease-modifying therapies have not been developed and approved. Integrins, in particular the αv and α3β1 integrins, as well as cadherins, in particular cadherin-11, appear to be important mediators of tissue fibrosis in multiple mouse models. Our understanding of how these adhesion molecules modulates the behavior of key cells that contribute to fibrosis, including the epithelial cell, macrophage, fibroblast and myofibroblast continues to expand. The targeting of αv and α3β1 integrins and cadherin-11 may allow more targeted inhibition of important fibrotic pathways such as TGFb and beta-catenin in the fibrotic tissue, where as general inhibtion of these pathways may result in too many unintended consequences. The translation of these observations into treatments is in various stages of development and clinical trials. Until then, additional insight into these pathways will continue to shed light into the development of tissue fibrosis and position us to translate these findings to the clinical arena as potential therapeutics for fibrotic diseases.

### Conflict of interest statement

Dr.Agarwal serves on the Scientific Advisory Board for Adheron Therapeutics and receives less than $10,000 for compensation.
